# Construction and application of prone position ventilation management scheme for severe COVID-19 patients

**DOI:** 10.3389/fphys.2023.1152723

**Published:** 2023-08-24

**Authors:** Xiuwen Chen, Cao Peng, Yao Xiao, Shiqing Liu

**Affiliations:** ^1^ Teaching and Research Section of Clinical Nursing, Xiangya Hospital, Central South University, Changsha, China; ^2^ Department of Operating Room, Xiangya Hospital, Central South University, Changsha, China; ^3^ Xiangya Nursing School, Central South University, Changsha, China; ^4^ National Clinical Research Center for Geriatric Disorders, Xiangya Hospital, Central South University, Changsha, China; ^5^ Department of Intensive Care Unit, Xiangya Hospital, Central South University, Changsha, China; ^6^ Department of Respiratory Medicine, Xiangya Hospital, Central South University, Changsha, China; ^7^ Xiangya Lung Cancer Center, Central South University, Changsha, China

**Keywords:** prone position ventilation, oxygenation index, nursing quality, management, COVID-19

## Abstract

**Background:** Prone position ventilation (PPV) can significantly improve oxygenation index and blood oxygen saturation in most (70%–80%) patients with acute respiratory distress syndrome. However, although PPV is not an invasive procedure, there are many potential PPV-related complications, such as nerve compression, crush injury, venous stasis (e.g., facial oedema), pressure sores, retinal damage, vomiting, and arrhythmia, with an incidence of up to 56.9%. Nursing managers have focused on reducing the occurrence of PPV-related complications and improving safety.

**Objective:** To construct a prone ventilation management scheme for patients with severe coronavirus disease 2019 (COVID–19) and analyse its application effect.

**Methods:** Based on a previous evidence-based study combined with the COVID-19 Diagnosis and Treatment Protocol (Trial Edition 9), a prone ventilation management protocol for severe COVID-19 was formulated and applied to COVID-19 patients in the intensive care unit of a designated hospital. A prospective self-control study was used to compare changes in the oxygenation index and other outcome indicators before and after the intervention.

**Results:** The oxygenation index of patients after intervention (321.22 ± 19.77 mmHg) was significantly higher (*p* < 0.05) than before intervention (151.59 ± 35.49 mmHg). The difference in oxygenation index in different prone position ventilation durations was statistically significant (*p* < 0.05). Nursing quality evaluation indicators showed that the implementation rate of gastric residual volume assessment was 100% and the incidence of occupational exposure and cross-infection was 0%; the incidences of pressure ulcers, drug extravasation, and facial oedema were 13.64% (3/22), 4.54% (1/22), and 4.54% (1/22), respectively. The incidence of unplanned extubation, aspiration, and falls/falls was 0%.

## 1 Introduction

Prone position ventilation (PPV), in which patients are mechanically ventilated from the prone position, was first developed in the 1970s as a way to improve the oxygenation method for acute respiratory distress syndrome (ARDS) (Petrone et al., 2021). Multiple randomised controlled studies have shown that prone ventilation can reduce the pleural pressure gradient of patients, restore ventilation in the dorsal segment of the lung, significantly improve the oxygenation index and blood oxygen saturation of patients, and reduce 28-day mortality (Munshi et al., 2017a; Douglas et al., 2021). Since the onset of the coronavirus disease 2019 (COVID-19), the outbreak has spread rapidly, leading to a global pandemic. In 2022, due to the characteristics of high infectivity, occultness, and fast transmission rate, the Omicron variant of the novel coronavirus circulating in Shanghai is widely susceptible to infection, especially in the older adults, who are prone to develop into severe and critical forms with high severity and fatality rates (Cai et al., 2022; Zhang et al., 2022). Sparing no effort to treat critically ill patients, improving the treatment rate, and reducing the case fatality rate has become important. Severe and critically ill patients should be treated in a standard prone position for no less than 12 h a day, according to the ninth version of the Diagnosis and Treatment Protocol for the novel coronavirus pneumonia. Therefore, standard and scientific PPV is the premise of ensuring the effectiveness of treatment and patient safety.

However, although PPV is not an invasive procedure, it is complex and has many potential complications. Several studies have shown that PPV can lead to complications such as stress injury, unplanned extubation, falls, aspiration, and arrhythmia, with an incidence as high as 56.9% (Malhotra and Kacmarek, 2020; Moore et al., 2020). In addition, Liu et al. (Liu et al., 2018) pointed out that the prone ventilation treatment rate of patients with severe ARDS in China was only 8.7%, and the high complication rate and low compliance were related to the lack of standardised surgical procedures. In recent years, clinical studies on PPV have mainly focused on the application effect of PPV in different populations and diseases and the analysis of its haemodynamic effect on patients (Huang et al., 2021; Lu et al., 2021). Nursing studies are mostly fragments of experience or summary, and there is still a lack of standard preventive measures and management plans for the whole process, not to say, related nursing guidelines or expert consensus to standardise the implementation of clinical PPV. Therefore, this study intended to develop management plans based on previous evidence-based PPV studies (Peng et al., 2021) and combined them with the characteristics of PPV in patients with severe novel coronavirus pneumonia to provide a basis and empirical reference for clinical nursing.

## 2 Methods

### 2.1 Construction of PPV management scheme for severe COVID-19

#### 2.1.1 Build a research team

The research team consisted of 12 members, including seven senior titles, three intermediate titles, one junior title, and one master’s degree student. It consisted of three experts in prone ventilation medicine, three in critical care medicine, one respiratory therapist, one evidence-based nursing expert, one scientific research nurse, two clinical nurses, and one graduate student. Specialists, respiratory therapists, and research nurses were primarily responsible for the development of management plans. Clinical nurses are responsible for personnel training, quality control, and program implementation. The research nurse was responsible for the overall project progress control and liaison consultations. The graduate students were responsible for collecting clinical data and outcome indicators. The research team regularly organised group meetings to implement the project’s progress, and the members worked closely with each other.

### 2.2 Develop a PPV management plan for severe COVID-19

This study was based on the previous PPV evidence-based research (Peng et al., 2021), combined with the Diagnosis and Treatment Protocol for Novel Coronavirus Pneumonia (Trial Ninth Edition). The management scheme of PPV was constructed from the aspects of standard operating flow, checklist, complication prevention, risk emergency plan, lung ultrasound-guided nursing flow and quality supervision, etc.

#### 2.2.1 Standard operating procedures

(1) Self-protection: Medical personnel should adopt protective measures in accordance with secondary protection standards.
(2) Medical evaluation: Evaluation of indications, contraindications, and informed consent. Indications: Common, severe, and critically ill patients with high-risk factors for severe COVID-19 and rapid disease progression. Contraindications: cervical spine injury, unstable fracture, heart surgery/post-traumatic thoracotomy within 24 h after heart surgery, severe haemodynamic instability, increased intracranial pressure, pregnancy, etc. cannot tolerate the prone position. Prior to prone ventilation, authorised informed consent was required.
(3) Nursing evaluation: Check the doctor’s orders and evaluate the patient’s vital signs, pipelines (arterial catheterisation, venous access, gastric tube, urinary tube, etc.), Richmond Agitation-Sedation Scale (RASS), gastric residual volume (suspension of enteral nutrient pumping, gastric residual volume), body weight, turning direction, and skin integrity.
(4) Materials: Prepare the rescue truck, turning sheet, closed sputum suction tube, electrode, extension tube, soft pillow, and pressure-relief tape.
(5) Personnel preparation: Every turn requires at least five teams of doctors, nurses, and respiratory therapists to work together, the role that team members introduce themselves and show, respiratory therapists, standing on the patient’s head to be responsible for the overall coordination, and patients with both need to have at least two staff, according to the number of patients with weight gain.
(6) Airway/respiration: The ventilator should be as close to the patient’s side as possible. A difficult airway intubation cart (bag) and negative pressure suction were placed in the standby state, and the results of laryngoscopy and length of endotracheal intubation were checked again. Fixation or binding of tracheal tubes; Oxygenate the patient with 100% oxygen; monitor tidal volume and inspiratory pressure; perform an arterial blood gas test, and record the results.
(7) Supine position to prone position: Start a timeout. Timeout refers to a pause before surgery. Perform a procedural check before the official turn to ensure that the team members are ready; try to stay away from the patient’s airway to reduce the risk of occupational exposure; loosen the cover, spread the rollover sheet, stick the decompression tape, prepare the head soft pillow; remove electrode sheet; fold the patient’s arm under the buttocks, palm facing forward, turn over the single wrap; turn over to the other side and stick the electrode sheet; the patient was turned over on the anteroposterior side with a single turn; head ring pad or face pad decompression, so that the patient’s arm in a swimming position, shoulder abduction 80°, elbow bending 90°; tidy the bed unit, cushion soft pillow, pay attention to privacy and heat preservation; keep head high and foot low, the head height of 30°; The length of prone position was recorded and each shift was handed over to continue treatment and nursing. The entire turning process was gentle, avoided large movements, and reduced the risk of aerosol transmission.
(8) Prone to supine position: Adjust the bed to a horizontal position, undress, and check the length of the catheter. Remove soft pillows. Turn to your side with your arms in front of you and wrap the rollover sheet. Subsequently, the electrode sheet was removed. An electrode sheet was attached to one side of the electrode. The patient returned to the supine position with a roll sheet. Clear respiratory tract, tidy bed unit, and record.

#### 2.2.2 Operation check sheet

A checklist for prone ventilation operations was developed and implemented as shown in Table 1.

**TABLE 1:** Checklist for prone position ventilation operations.

Project	Content of verification													Results of verification	Signature
1	Evaluate and check doctor’s orders														
2	Materials: 3 soft pillows, 1 silicone pillow, 2 nursing pads, 1 medium sheet, 1 set of closed sputum suction tubes, 5 ECG electrodes, 3 cotton pads, intubation cart (bag) and rescue cart														
3	Patient preparation: intravenous access, ECG monitoring, indwelling gastric tube and urinary tube, an indwelling catheter for measuring pressure, ensure that all tubes are long enough and properly fixed														
4	Check catheter position, stop nasal feeding, evacuate gastric residual, determine turning direction, administer pure oxygen, record ventilator parameters														
5	Team members take the position, release the electrodes, cuff, and gown, move the patient to the edge of the bed (proximal side), extend the distal arm naturally, and press it under the ipsilateral hip														
6	Place a soft pillow on the abdomen and chest, cover the chest of the patient with a medium sheet, wrap the distal arm, and press them together under the patient														
7	Put the patient in the lateral decubitus position, pull the medium sheet edge under the distal hand horizontally, and another team member pulls the proximal side medium sheet edge to turn the patient over and move the patient to the centre. The member standing on the head protects the patient’s cervical vertebra and maintains the pipeline unobstructed, and connects the ECG monitoring														
8	Maintain the patient’s upper body breaststroke position with the right hand slightly raised and the right leg slightly bent to promote comfort and protect the skin														
9	Adjust the bed to head high and feet low, clean up the secretions from the mouth and nose, arrange the pipes, and properly fix it														
10	Arrange the bed unit, record the vital signs and adjust the parameters of the ventilator														
11	Assist the patient to turn his face to the opposite side every 2 h, change the position of hands and feet at the same time, suction sputum as needed, slow nasal feeding, and close observation														
	Direction	R	L	R	L	R	L	R	L	R	L	R	L		
	Time														
	Signature														
12	At the end of the prone position, arrange all the pipes, remove the electrodes, take off the hospital gown, remove all pillows under the patient, and place both hands and feet on the floor until completely supine														
13	The person standing at the head fixed the breathing line and endotracheal tube and maintained the functional position of each line to prevent slip. The team members moved the patient to face the side of the bed, put the far arm under the patient, held the opposite medium sheet, supported the shoulder and hip, and synchronously turned the patient back to a supine position														
14	Intimate electrodes put on a gown, record vital signs														
15	Clean up the secretions from the mouth and nose, arrange the pipes and fix them properly														
Note															

#### 2.2.3 Complication prevention and risk emergency plan

According to the 6s model (Xie et al., 2021) of evidence-based resources from the top down to the principle of computer retrieval PPV-associated complication prevention guidelines, system evaluation, project, etc., evidence extraction prevents stress damage (Malhotra and Kacmarek, 2020; Moore et al., 2020; Peng et al., 2021), planned extubation (Intensive Care Society and Faculty of Intensive Care Medicine, 2019; Tissue Viability Society, 2020), and facial oedema (Intensive Care Society and Faculty of Intensive Care Medicine, 2019; Tissue Viability Society, 2020), evidence of common complications such as aspiration (Intensive Care Society and Faculty of Intensive Care Medicine, 2019) (Table 2), and the brainstorming method is used to formulate the corresponding risk contingency plans. Meanwhile, according to the characteristics of the transmission of the novel coronavirus, an occupational exposure disposal system and procedures for medical staff in the isolation ward of the novel coronavirus were formulated.

**TABLE 2:** Summary of evidence on the prevention of complications related to prone position ventilation.

Complications	Content of evidence	Recommended level
Stress-induced injury	It is recommended to evaluate the risk of stress-induced injury before prone position ventilation (Moore et al., 2020)	A
	Regularly assess high-risk areas in the prone position, including cheeks, auricle, clavicle, chest, breast, pubic symphysis, iliac crest, male genitalia, knees and toes. To assess changes in skin and tissue integrity, colour, temperature, hardness and humidity, and to assess the grading of pressed red or damaged skin (Malhotra and Kacmarek, 2020; Peng et al., 2021)	A
	It is recommended to prophylactically apply transparent film dressing, foam dressing, hydrocolloid dressing, etc., in high-risk or key compression parts (Malhotra and Kacmarek, 2020; Peng et al., 2021)	A
Unplanned extubation	A special person is responsible for tube management during a position change. For patients who receive extracorporeal membrane oxygenation (ECMO) treatment, it is recommended that an additional person be assigned to manage the ECMO tube during the process of turning over (Intensive Care Society and Faculty of Intensive Care Medicine, 2019)	B
	Ensure that all conduits are reserved for adequate length, and use extension tubing if necessary (Tissue Viability Society, 2020)	A
	It is suggested to have double fixation of the tube and reasonably restrain the patient (Intensive Care Society and Faculty of Intensive Care Medicine, 2019)	B
Oedema of the face	The head direction should be changed every 2 h, the head ring pad or facial pad should be used for decompression, and the protective pad with adsorption function should be placed under the head to reduce the stimulation of oral or nasal secretions on the skin (Intensive Care Society and Faculty of Intensive Care Medicine, 2019; Tissue Viability Society, 2020)	A
	The patient’s eyes are closed and protected with gauze or film to ensure that the eyelashes face outward to avoid direct eye pressure (Intensive Care Society and Faculty of Intensive Care Medicine, 2019)	A
	In the prone position, the head is high and the feet are low, and the head of the bed is kept at 30° to reduce head and face oedema (Intensive Care Society and Faculty of Intensive Care Medicine, 2019)	A
Aspiration	Before the position change, the enteral nutrient solution pumping was suspended, and the gastric residual volume was evacuated (Intensive Care Society and Faculty of Intensive Care Medicine, 2019)	A
	It is recommended to evaluate gastric residual volume in each class (Intensive Care Society and Faculty of Intensive Care Medicine, 2019)	B

#### 2.2.4 Nursing process guided by lung ultrasound

Lung re-expansion in the gravity-dependent area was evaluated using severe ultrasound to guide the treatment time and frequency of prone ventilation. At the same time, the effectiveness of prone ventilation was predicted by the semi-quantitative lung ultrasound score, and *in vitro* treatment, turning over, back-patting, and mechanical sputum drainage were also guided. In addition, prone ventilation often requires deep sedation or muscle relaxation therapy, which may affect circulation. In this study, the team used ultrasound to evaluate and monitor the haemodynamics of patients and selected the appropriate cardiac output for patients, thus playing a role in protecting pulmonary circulation.

#### 2.2.5 Quality supervision

The Donabedian structure-process outcome model was adopted as the theoretical framework, and two rounds of the Delphi method of expert correspondence consultation were used to determine the evaluation indices of prone ventilation nursing quality in patients with severe novel coronavirus pneumonia. Quality supervision was carried out on the entire prone ventilation process of patients with severe novel coronavirus pneumonia from three dimensions: structure, process, and result.

## 3 Clinical application of prone ventilation management scheme in patients with severe novel coronavirus pneumonia

### 3.1 Sample

In April 2022, under the deployment of the National Health Commission and Shanghai Municipal Health Commission, the third batch of medical teams from Hunan Province took over the medical treatment, nursing, infection prevention, and control work of the intensive care unit (ICU) of a designated hospital for treating COVID-19 in Shanghai. This study included patients with severe novel coronavirus pneumonia who were admitted to the ICU as the research object. Inclusion criteria were as follows: 1) age >18 years; 2) any of the following:① shortness of breath, RR ≥ 30 times/min; ② in the resting state, the oxygen saturation <93%; ③ partial pressure of oxygen (PaO_2_)/concentration of oxygen (FiO_2_) ≤ 300 mmHg (1 mmHg = 0.133 kPa); ④progressive aggravation of clinical symptoms, lung imaging shows an obvious progression of the lesion >50% within 24–48 h; 3) the time of prone position ventilation being intubated ≥12 h; 4) patients or family members signed informed consent. The sample size was calculated using the method of estimating the sample size of the paired design, n = [(α+β)σd/δ]^2, where *δ* is the required differentiation, *σd* is the population standard deviation of each pair difference. Referring to the previous study (Douglas et al., 2021), where the oxygenation index σd was 147 and *δ* was 107, the sample size was calculated to be 16, and a 10% loss of follow-up was considered. Therefore, the required sample size was 19.

### 3.2 Research methods

A prospective self before and after the control study was used to manage the subjects meeting the inclusion criteria strictly in accordance with the prone position ventilation management scheme for severe novel coronavirus pneumonia, and the changes in various outcome indicators before and after intervention were compared to evaluate the effect. To ensure the homogeneity of intervention quality, all members of the medical team were trained by two specialist nurses in the form of Tencent conferences, face-to-face meetings, and operation demonstrations. The training content covers management schemes such as the standard operating flow of prone ventilation, checklist, complication prevention, risk emergency plan, lung ultrasound-guided nursing flow, and quality supervision. Nursing personnel could only participate in the study after they passed the assessment.

### 3.3 Effect evaluation index and data collection method

The oxygenation index, also known as the ventilation/perfusion index, was calculated as arterial oxygen partial pressure (PaO_2_)/oxygen absorption concentration (FiO_2_) ×100%. In this study, the oxygenation index of patients was obtained by blood gas extraction before prone position ventilation, 8, 12, and 16 h after treatment to evaluate the changes in oxygenation before and after prone position ventilation. At the same time, the incidence of occupational exposure, incidence of cross-infection, execution rate of gastric residual volume assessment, incidence of stress injury, incidence of unplanned extubation, incidence of facial oedema, aspiration, drug exosmosis, and fall/fall incidence were analysed.

### 3.4 Statistical methods

SPSS 22.0 software was used for data entry and statistical analysis. The measurement data were described by mean ± SD and analysed using the t-test or variance analysis. The data were described by frequency and percentage and analysed statistically by chi-square test, Fisher’s exact probability test, or rank sum test. Statistical inference was performed according to a test level of α = 0.05. The *p*-value was a bilateral probability value, and *p* < 0.05 meant that the difference was statistically significant.

## 4 Results

### 4.1 General data analysis

A total of 22 severely ill patients with prone ventilation were included, including nine males and 13 females. There were 2 cases of the normal type, 15 cases of the severe type, and 5 cases of the critical type. The patients were aged between 68 and 96 (85.82 ± 8.20) years, and the RASS score before prone ventilation was −5 to −3 (−4.41 ± 0.59) points. Acute physiology and chronic health evaluation Ⅱ (APACHE Ⅱ) was 13–23 (19.05 ± 2.34) minutes, the total duration of prone ventilation was 19.00–52.00 (34.14 ± 8.22) h, an average of 12.67–22.00 (16.83 ± 2.46) h per day. In addition to the novel coronavirus pneumonia, admission diagnosis also included hypertension, diabetes, coronary heart disease, sequelae of cerebral infarction, cirrhosis, epilepsy, sequelae of intracerebral haemorrhage, intracerebral haemorrhage in the basal ganglia area breaking into the ventricle, and renal failure (Supplementary Table S1).

### 4.2 Trend of oxygenation index before and after prone ventilation treatment

The oxygenation index of patients with severe novel coronavirus pneumonia after intervention with the prone ventilation management scheme (321.22 ± 19.77 mmHg) was significantly higher than that before intervention (151.59 ± 35.49 mmHg), and the difference was statistically significant (*p* < 0.05). A comparison of the oxygenation index of ventilation duration in different prone positions is shown in Table 3 **,** and the results show that with an increase in ventilation duration in the prone position, the oxygenation index of patients showed a significant linear growth trend (*p* < 0.05).

**TABLE 3:** Trend of oxygenation index before and after prone position ventilation (PPV) treatment (n = 22).

	Before PPV treatment	PPV treatment for 8 h	PPV treatment for 12 h	PPV treatment for 16 h	After PPV treatment	*F*	*P*
Oxygenation index (mmHg)	151.59 ± 35.49	203.72 ± 38.65	264.95 ± 34.13	315.31 ± 21.05	321.22 ± 19.77	206.707	0.000

### 4.3 Analysis of nursing quality evaluation index

Twenty-two patients were turned from the supine to the prone position 45 times. The implementation rate of the gastric residual volume assessment before position conversion was 100%. The incidence of occupational exposure and cross-infection during the operation for position conversion was 0%. The incidence rates of ventilation-related complications in the prone position were 13.64% (3/22) for pressure injury, 4.54% (1/22) for drug exosmosis, 4.54% (1/22) for facial oedema, and 0% for unplanned extubation, aspiration, and fall/fall.

## 5 Discussion

### 5.1 The oxygenation index of patients with severe novel coronavirus pneumonia varies with the time of prone position

The lungs of patients with severe novel coronavirus pneumonia mainly exhibit bilateral diffuse alveolar injury with fibrous mucinous exudation. Patients with ARDS experience hypoxia, which can lead to multiple organ dysfunction and even death. Therefore, reducing hypoxia is a key factor in the treatment of COVID-19 patients. Several multi-centre, prospective, randomised controlled studies (Guérin et al., 2013; Munshi et al., 2017b) have shown that prone ventilation can not only improve the oxygenation of patients but also significantly reduce the mortality of 28 and 90 days for patients with severe ARDS by extending prone ventilation at an early stage. In this study, prone ventilation was used as the main measure to “advance the threshold” in the treatment of severe novel coronavirus pneumonia. The early intervention significantly improved the oxygenation index of patients with severe novel coronavirus pneumonia, and the average oxygenation index was greater than 300 mmHg, which was highly recognised by experts in the medical treatment group of the Joint Prevention and Control Mechanism of The State Council. It was also introduced and promoted in the training sessions of designated and makeshift hospitals in Shanghai, which confirmed the importance of prone ventilation in the treatment of novel coronavirus pneumonia. In addition, the results of this study showed that there were significant differences in the oxygenation index of patients with severe novel coronavirus pneumonia with different prone position ventilation durations, and with the increase in prone position ventilation duration, the oxygenation index of patients showed a significant linear growth trend. Therefore, it is important to standardise and implement the length of the prone position in the treatment of prone ventilation. It is recommended that all designated hospitals strictly comply with the Diagnosis and Treatment Protocol for Novel Coronavirus Pneumonia (Trial Version 9) and ensure that the prone position is treated for at least 12 h per day. The 2019 International Guidelines Official Guidelines: Management of Acute Respiratory Distress Syndrome (Papazian et al., 2019) strongly recommends that ARDS patients with an oxygenation index less than 150 mmHg should continue prone ventilation for at least 16 h. In this study, the average duration of prone ventilation for patients with severe novel coronavirus pneumonia was 16.83 h per day. This also indicates that the implementation of the management scheme in this study improves compliance with prone ventilation therapy to a certain extent.

### 5.2 The prone position ventilation management scheme for severe novel coronavirus pneumonia can standardise the implementation of clinical PPV

This study, according to the results of prone position ventilation in patients with gastric residual position prior to the conversion of quantitative evaluation are enforced is 100%. The incidence of complications related to aspiration-prone position ventilation was 0%, suggesting that the correct nursing assessment is the precondition for the implementation of prone position ventilation, evaluation, and risk assessment in addition to skin prone position ventilation duct outside the risk assessment which still needs to focus on assessing gastric residual volume and reducing the incidence of complications, such as aspiration. Because of the new coronavirus infectious pneumonia, we have put the severe new coronavirus pneumonia nursing quality evaluation index in prone position ventilation and invited fixed-point hospital joint experts to write to the circuit court sense of experts. This has increased the incidence of occupational exposure and cross-infection incidence of outcome indicators, and the results showed that the incidence was 0%, suggesting that the implementation of this program has strengthened the implementation of infection prevention and control measures. Second, according to the results of prone position ventilation duct complications related to the highest stress injury, has to do with Moore (Moore et al., 2020), such as the results are consistent, analyse the causes, and possible thinning and senile patients with skin and associated with basic diseases such as diabetes; further prompt medical personnel need to be strengthened in the prone position treatment in patients with skin protection. It is recommended to use the Braden stress injury risk scale for daily assessment, and formulate corresponding nursing measures according to the assessment results. The Omicron variant of the novel coronavirus circulating in Shanghai is highly contagious, and most severe novel coronavirus pneumonia cases occur in older adults patients. The average age of severe patients included in this study was 85.82 years, and most of them were complicated with basic diseases such as hypertension, diabetes, coronary heart disease, and cerebral infarction, most of whom were physically disabled, which brought great challenges to treatment and nursing. But this study through the formulation and application of severe new coronavirus pneumonia patients prone position ventilation management scheme, to the largest extent, reduces the prone position ventilation related nursing security incidents, visible, severe new coronavirus pneumonia patients prone position ventilation management consultation can standardise the prone position ventilation operation process, operation checklist and nursing quality evaluation index. It is beneficial to improve nursing quality and promote patient safety.

## Conclusion

This study was based on previous evidence-based research, combining the Chinese diagnosis and treatment plan for COVID-19 (Trial Version 9), from the prone position ventilation operation flow, operation checklist, complications, risk prevention, emergency plan, lung ultrasound guidance of nursing process, and quality supervision critical aspects such as building a new coronavirus pneumonia prone position ventilation management solution. The application of this scheme can standardise and promote the implementation of PPV for severe novel coronavirus pneumonia, improve the quality of care, and improve the prognosis of patients. The evaluation index of nursing quality can facilitate a more objective and comprehensive clinical evaluation of the nursing quality of prone position ventilation for severe novel coronavirus pneumonia, and achieve continuous quality improvement. However, this study has the following limitations: 1) small sample size; 2) given ethical reasons, no control group was set up; only before and after the study subjects themselves were compared. Because the severity of the disease is not completely consistent in the comparison, it is difficult to ensure consistency of the starting points of the two stages, which may affect the comparability of the two stages. Therefore, the results of this study need to be further verified.

## Data Availability

The original contributions presented in the study are included in the article/Supplementary Material, further inquiries can be directed to the corresponding author.
