# Supplementary material for: Construction and application of prone position ventilation management scheme for severe COVID-19 patients
Source: Front Physiol. 2023 Aug 24;14:1152723. doi: 10.3389/fphys.2023.1152723 (PMC10488700; doi:10.3389/fphys.2023.1152723)
Supplement: Supplementary file 1 [file Presentation1.pdf]

Table S1. Characteristics of patients

Case	Sex	Types	Age (years)	Admission diagnosis	RASS score	APACHE Ⅱ score	The total duration of prone ventilation (h)	The duration of prone ventilationan per day (h)
1	F	Severe	95	Novel coronavirus pneumonia; Hypertension; Coronary heart disease	-4	21	48	16
2	M	Severe	88	Novel coronavirus pneumonia; Hypertension; Diabetes	-5	22	26	17
3	M	Severe	86	Novel coronavirus pneumonia; Sequelae of cerebral infarction	-4	23	52	17
4	F	Normal	82	Novel coronavirus pneumonia; Hypertension; Renal failure	-5	19	36	18
5	F	Severe	74	Novel coronavirus pneumonia; Sequelae of intracerebral haemorrhage	-4	15	22	15
6	F	Critical	96	Novel coronavirus pneumonia; Intracerebral haemorrhage in the basal ganglia area breaking into the ventricle	-4	21	19	19
7	F	Severe	92	Novel coronavirus pneumonia; Cirrhosis	-5	19	37	14
8	M	Severe	90	Novel coronavirus pneumonia; Epilepsy	-3	18	32	21
9	M	Severe	93	Novel coronavirus pneumonia; Coronary heart disease; Sequelae of cerebral infarction	-5	17	34	17
10	F	Critical	77	Novel coronavirus pneumonia; Hypertension; Diabetes	-5	18	28	19
11	M	Severe	92	Novel coronavirus pneumonia; Intracerebral haemorrhage in the basal ganglia area breaking into the ventricle	-5	19	33	17
12	F	Severe	94	Novel coronavirus pneumonia; Cirrhosis; Diabetes	-5	21	36	18
13	M	Critical	89	Novel coronavirus pneumonia;	-4	18	35	18

				Coronary heart disease				
14	M	Severe	79	Novel coronavirus pneumonia;<br>Hypertension;<br>Diabetes	-4	19	29	14.50
15	M	Severe	89	Novel coronavirus pneumonia;<br>Diabetes;<br>Coronary heart disease	-4	20	28	18.67
16	F	Critical	68	Novel coronavirus pneumonia;<br>Sequelae of cerebral infarction;<br>Hypertension	-4	18	43	14.33
17	F	Severe	74	Novel coronavirus pneumonia;	-5	19	38	12.67
18	F	Severe	91	Novel coronavirus pneumonia;<br>Sequelae of intracerebral haemorrhage	-4	21	36	18.00
19	F	Critical	90	Novel coronavirus pneumonia;<br>Diabetes	-5	13	40	13.33
20	M	Severe	88	Novel coronavirus pneumonia;<br>Cirrhosis	-4	17	22	22.00
21	F	Severe	89	Novel coronavirus pneumonia;<br>Diabetes;<br>Coronary heart disease	-5	19	42	14.00
22	F	Normal	72	Novel coronavirus pneumonia;<br>Hypertension	-4	22	35	17.50

RASS, Richmond Agitation-Sedation Scale; APACHE Ⅱ, Acute physiology and chronic health evaluation; F, female; M, male.
